# Autocrine Production of β-Chemokines Protects CMV-Specific CD4^+^ T Cells from HIV Infection

**DOI:** 10.1371/journal.ppat.1000646

**Published:** 2009-10-30

**Authors:** Joseph P. Casazza, Jason M. Brenchley, Brenna J. Hill, Ribka Ayana, David Ambrozak, Mario Roederer, Daniel C. Douek, Michael R. Betts, Richard A. Koup

**Affiliations:** 1 Immunology Laboratory, Vaccine Research Center, NIAID, NIH, Bethesda, Maryland, United States of America; 2 Immunopathogenesis Unit, Lab of Molecular Microbiology, NIAID, NIH, Bethesda, Maryland, United States of America; 3 ImmunoTechnology Section, Vaccine Research Center, NIAID, NIH, Bethesda, Maryland, United States of America; 4 Human Immunology Section, Vaccine Research Center, NIAID, NIH, Bethesda, Maryland, United States of America; 5 Department of Microbiology, University of Pennsylvania, Philadelphia, Pennsylvania, United States of America; University of Zurich, Switzerland

## Abstract

Induction of a functional subset of HIV-specific CD4^+^ T cells that is resistant to HIV infection could enhance immune protection and decrease the rate of HIV disease progression. CMV-specific CD4^+^ T cells, which are less frequently infected than HIV-specific CD4^+^ T cells, are a model for such an effect. To determine the mechanism of this protection, we compared the functional response of HIV gag-specific and CMV pp65-specific CD4^+^ T cells in individuals co-infected with CMV and HIV. We found that CMV-specific CD4^+^ T cells rapidly up-regulated production of MIP-1α and MIP-1β mRNA, resulting in a rapid increase in production of MIP-1α and MIP-1β after cognate antigen stimulation. Production of β-chemokines was associated with maturational phenotype and was rarely seen in HIV-specific CD4^+^ T cells. To test whether production of β-chemokines by CD4^+^ T cells lowers their susceptibility to HIV infection, we measured cell-associated Gag DNA to assess the *in vivo* infection history of CMV-specific CD4^+^ T cells. We found that CMV-specific CD4^+^ T cells which produced MIP-1β contained 10 times less Gag DNA than did those which failed to produce MIP-1β. These data suggest that CD4^+^ T cells which produce MIP-1α and MIP-1β bind these chemokines in an autocrine fashion which decreases the risk of *in vivo* HIV infection.

## Author Summary

HIV infection results in a significant loss of CD4^+^ T cells, particularly HIV-specific CD4^+^ T cells. In contrast to this, CMV-specific CD4^+^ T cells persist in large numbers, even in individuals with AIDS. We compared the functional profile of HIV-specific and CMV-specific CD4^+^ T cells and found that unlike HIV-specific CD4^+^ T cells, CMV-specific CD4^+^ T cells rapidly produce MIP-1β when stimulated with cognate antigen. CMV specific CD4^+^ T cells also produce another β chemokine when stimulated with cognate antigen, MIP-1α. Addition of both of these chemokines to *in vitro* incubations protects CD4^+^ T cells from HIV infection. To determine if the production of these two chemokines could protect the CD4^+^ T cells that produce them *in vivo*, we analyzed peripheral blood cells from HIV infected individuals and separated CMV-specific CD4^+^ T cells that produced MIP-1β from CMV-specific CD4^+^ T cells that did not. We found that cells that produced MIP-1β were less frequently infected with HIV than those that did not produce MIP-1β. These data, and recent advances in vaccine design, suggest that it may be possible to design a vaccine in which vaccine-induced HIV-specific CD4^+^ T cells are less susceptible to infection than those usually produced during HIV infection.

## Introduction

Antigen-specific CD4^+^ T cells are thought to play an important role in control of HIV and CMV infections. Strong CD4^+^ proliferative responses to p24 are associated with control of viremia and maintenance of cytotoxic T-lymphocytes (CTL) in HIV-infection [1],[2]. Plasma HIV load is inversely correlated with the frequency of p24-specific IL-2 secreting CD4^+^ T cells [3]. Data from transplant studies suggest that CMV-specific CD4^+^ T cells are required to maintain CMV-specific CD8^+^ T cells [4]. The development of CMV disease in HIV infected individuals is associated with the loss of CMV-specific CD4^+^ T cells [5]–[7]. Recovery of effective CMV immunity after initiation of highly active antiretroviral therapy also appears to occur concurrently with recovery of CMV-specific CD4^+^ T cells [7],[8].

While HIV-specific CD4^+^ T cells are preferentially infected and depleted by HIV, CMV-specific CD4^+^ T cells are often easily identifiable even in late stage HIV [9]–[11] and evidence of CMV disease does not occur until endstage AIDS when CD4^+^ T cells counts are <100 and more often <50 cell/µl [12],[13]. This suggests that CMV-specific CD4^+^ T cells may produce factors that protect them from HIV infection *in vivo*. Delineating these factors may provide clues as to the types of HIV-specific CD4^+^ T cells one would hope to engender with an effective HIV vaccine.

CMV-specific CD4+ T cells rapidly produce MIP-1β when stimulated by their cognate antigen [14]. Binding of MIP-1β or the related chemokines MIP-1α and RANTES can protect CD4^+^ T cells from infection by CCR5-tropic (R5) HIV [15]–[17]. MIP-1α, MIP-1β and RANTES all bind human CCR5 at sub-nanomolar levels [18]. Binding of these ligands to CCR5 could protect against HIV infections by two mechanisms: i) blocking of the receptor binding site for HIV, and ii) downregulating surface expression of CCR5 [19]. Although protection of CD4^+^ T cells from HIV-infection *in vitro* has been shown by the exogenous addition of MIP-1α, MIP-1β and RANTES to CD4^+^ T cells in culture [20]–[22], by the production of MIP-1α, MIP-1β and RANTES by CD8^+^ T cells cultured with CD4+ T cells [21],[23],[24], and by the production of MIP-1α, MIP-1β and RANTES by CD4^+^ T cells themselves [25],[26], little direct evidence exists showing that production of these chemokines actually protect CD4^+^ T cells from infection *in vivo*.

Here we assess the production of cytokines, chemokines, and their respective mRNAs by CMV-specific CD4^+^ T cells. We use cell-associated Gag DNA to assess the HIV infection history of comparable antigen-specific CD4^+^ T cells which either do or do not produce MIP-1β. Our data demonstrate that β-chemokine production by antigen-specific CD4^+^ T cells is associated with a ten-fold reduction in HIV infection *in vivo*.

## Results

### CMV-specific CD4^+^ T cells are more polyfunctional than HIV-specific CD4^+^ T cells

**Figure 1:**
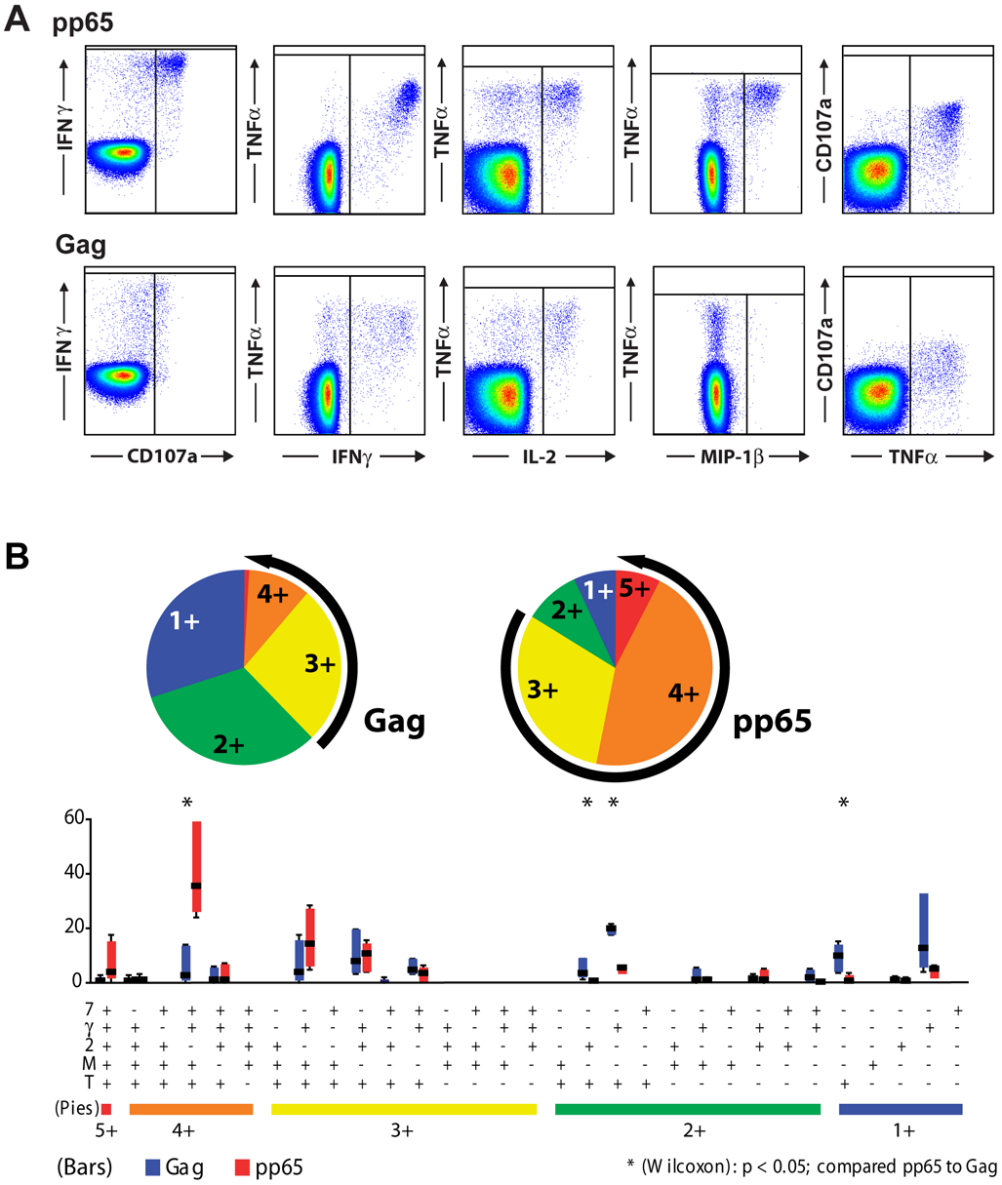
Comparison of polyfunctionality of gag-specific and pp65-specific CD4^+^ T cell responses. A) Representative plots showing functional responses to pp65 15mers overlapping by 11, and gag 15mers overlapping by 11. B) Individual pie charts show the percentage of antigen-specific CD4^+^ T cells which produce 1, 2, 3, 4, or 5 functional responses for gag- and pp65-specific CD4^+^ T cells. Three, four, and five functional responses are highlighted by the black arrows. Polyfunctional responses were significantly more frequent in pp65-specific CD4^+^ T cells than in gag-specific CD4^+^ T cells (*P*<0.05). Frequency of functional species for pp65-specific (red bars) and Gag-specific (blue bars) responses are shown in the bar graph for each of the 31 functional species. Colored bars show the range for the 2^nd^ and 3^rd^ quartiles, medians are denoted by the horizontal bar and standard deviations are shown by whiskers. Significant differences between the frequencies of different individual gag- and pp65-specific functional species are indicated by a * (*P*<0.05).

We identified six individuals, not on antiretroviral therapy, with comparable magnitude of antigen-specific responses to overlapping CMV pp65 and HIV gag peptides (Table 1). The median CMV response was 0.87% (range 0.15–2.38%) and the median HIV response was 0.52% (0.24–1.61%). Although the frequencies of HIV-specific CD4^+^ T cells and CMV-specific CD4^+^ T cells were not significantly different in these individuals, their patterns of response were markedly different (Figure 1A). Of the five functions measured, (production of IFNγ, IL-2, MIP-1β and TNFα, and a marker of degranulation, surface mobilization of CD107a), the average HIV-specific T cell exhibited 2.1 (1.8–2.6) different functions whereas the average CMV-specific T cell was more polyfunctional exhibiting 3.4 (2.9–3.6) different functions (***P***<0.05). Production of IFNγ, MIP-1β, TNFα and mobilization of CD107a was the most common functional combination observed in CMV-specific CD4^+^ T cells, comprising a median of 47% (22–62%) of CMV-specific cells. This functional combination comprised only 2% (1–19%) of the total HIV-specific response (Figure 1B). Instead, CD4^+^ T cells producing only IL-2 and TNFα, IFNγ and TNFα, or TNFα alone made up most of the total HIV-specific response. The median percentages of CMV-specific CD4^+^ T cells producing IFNγ (98%), TNFα (88%), and IL-2 (27%), were similar to or marginally higher than, the median percentages of HIV-specific CD4^+^ T cells producing these cytokines (85%, 64%, and 29% respectively). The main functional difference between HIV- and CMV-specific CD4^+^ T cells was defined by production of MIP-1β and mobilization of CD107a; the median percentages of CMV-specific CD4^+^ T which mobilized CD107a (58%) and produced MIP-1β (63%) were much greater than in HIV-specific CD4^+^ T cells (28% and 12%, respectively, ***P***≤0.05).

**Table 1:** Age, Sex, viral load, CD4 count and total Gag and pp65-specific CD4^+^ responses of subjects studied.

Subject	VL	CD4	Age (Sex)	Experiment*	Gag§ response	pp65§ response
H1	250	510	62 (M)	A	0.71	1.3
H2	553	656	52 (M)	A,	1.61	0.54
H3	215	380	58 (M)	A	0.78	1.36
H4	477	421	40 (M)	A,	0.23	0.24
H5	776	395	35 (M)	A, D, E	0.36	0.15
H6	<50	347	40 (M)	A, D, E	0.79	2.38
H7	188000	603	39 (M)	B		1.62
H8	6210	331	48 (M)	B		14.1
H9	834	113	45 (F)	B, C		0.76
H10	669	133	59 (M)	C		1.25
H11	251	257	42 (F)	C, D		3.33
H12	35300	145	46 (F)	D		0.39
H13	15300	752	29 (M)	D, E		0.14
H14	3100	430	23 (M)	D, E		0.2
H15	68500	328	35 (M)	D, E		8.7
H16	5300	448	46 (M)	E		0.12
H17	14500	272	21 (M)	E		0.45
H18	4410	685	33 (M)	E		1.69
C1			46 (M)	B		1.51
C2			52 (M)	B		1.91
C3			37 (F)	B, C		2.97
C4			44 (M)	C		0.89
C5			61 (M)	C		2.14
C6			23 (M)	D		0.94
C7			51 (M)	D		1.2
C8			33 (M)	C		0.5
C9			43 (M)	D		0.57
C10			25 (M)	D		0.29
C11			45 (M)	D		0.17
C12			43 (M)	D		2.87

**Figure 2 ppat-1000646-g002:**
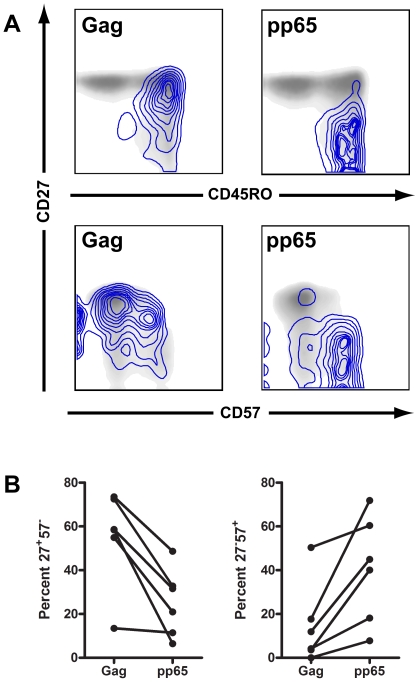
pp65-specific CD4^+^ T cells are more mature than Gag-specific CD4^+^ T cells. A) Gag-specific and pp65-specific CD4^+^ T cells, shown by blue isobars, are overlaid onto 2 dimensional density plots for total CD4^+^ T cells plotted against CD27 and CD45RO or CD27 and CD57 surface expression. B) For each of the 6 individuals characterized gag-specific CD4^+^ T cells had higher CD27 expression and lower CD57 responses than did pp65-specific CD4^+^ T cells (*P*<0.05).

*(A) indicates PBMC used for the functional analysis shown in Figure 1, 2, and 3. (B) indicates PBMC used for the determination of the production of MIP-1α, MIP-1β, RANTES, and IFNγ shown in Figure 4A. (C) indicates PBMC used for the determination of relative amounts of MIP-1α, MIP-1β, RANTES, and IFNγ mRNA shown in Figure 4B. (D) indicates PBMC used for the determination of surface CCR5 expression shown in Figure 5. (E) indicates PBMC used for the determination of cell associated Gag DNA shown in Figure 6.

**§:** Gag and pp65 responses are given as a percent of the total CD4^+^ population.

Although the median polyfunctionality of CMV-specific CD4^+^ T cells was greater than that found in HIV-specific CD4^+^ T cells, the hierarchy of the functional responses were similar for CD4^+^ T cells with similar levels of polyfunctionality (Figure 1B). For instance, in both HIV- and CMV-specific CD4^+^ T cells, those which produced only one response usually produced either IFNγ or TNFα. Cells which showed two different functional responses usually produced either IFNγ and TNFα although some cells produced IL-2 instead of IFNγ or TNFα. Cells which produced three functional responses almost always produced IFNγ and TNFα with either IL-2, MIP-1β or mobilized CD107a in response to antigenic stimulation. Most cells which produced four different functional responses lacked only IL-2.

### CMV-specific CD4^+^ T cells are more mature than HIV-specific CD4^+^ T cells

There are several reports that CMV-specific CD4^+^ T cells are more mature than HIV-specific CD4^+^ T cells as defined by expression pattern of CCR7, CCR5, CD27, CD28 and CD57 [3],[9],[27],[28]. We therefore assessed the proportion of the HIV- and CMV-specific responses that expressed the surface markers CD45RO, CD27, and CD57 (Figure 2A and 2B). CMV-specific CD4^+^ T cells were less frequently CD27^+^CD45RO^+^, median 26% (6–49%), than HIV-specific CD4^+^ T cells, median 58% (13–74%) (***P***<0.05). CMV-specific CD4^+^ T cells were also more frequently CD27^−^CD57^+^, median 42% (8–72%), than HIV-specific CD4^+^ T cells, median 15% (0–50%) (***P***<0.05).

Unlike HIV-specific CD8^+^ T cells in which CD57 positivity was frequently seen on CD27^+^ cells, almost all CD57^+^ CMV positive CD4^+^ T cells were CD27^−^ (Figure 2A) suggesting an orderly maturational pathway with memory CD4^+^ T cells first expressing CD45RO and CD27, then CD45RO alone, and then CD45RO and CD57. The majority of CMV specific CD4^+^ T cells were CD27^−^57^+^, median 42% (8–72%), followed by CD27^−^CD57^−^, median 30% (21–48%) and CD27^+^CD45RO^+^, median 26% (6–49%).

### The functional pattern of CMV-specific and HIV-specific CD4^+^ T cells changes with maturation

**Figure 3:**
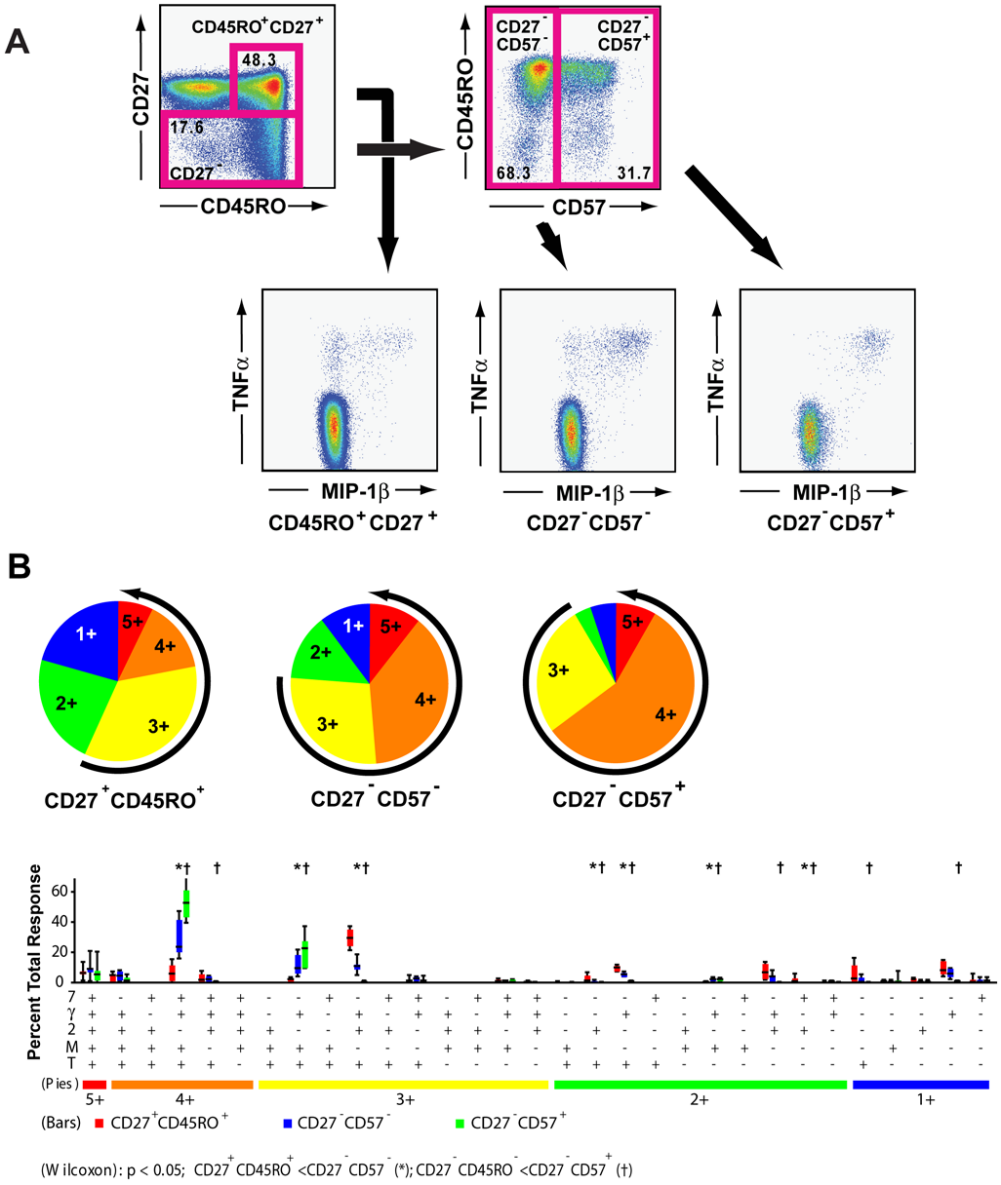
pp65-specific CD4^+^ T cells become more polyfunctional with increasing maturity. A) Representative plots showing sorting of CD4^+^ T cells into CD27^+^CD45RO^+^, CD27^−^CD57^−^ and CD27^+^CD57^+^ populations and their respective staining for TNFα and MIP-1β. The percentage of CMV-specific CD4^+^ T cells which produced both TNFα and MIP-1β increased dramatically with increasing maturation. B) Maturation markedly increased polyfunctionality of pp65-specific CD4^+^ T cells. Percentage of the total response for CD27^+^CD45RO^+^ (red bars), CD27^−^ CD57^−^ (blue bars) and CD27^−^CD57^+^ (green bars) memory CD4^+^ T cells is shown for each of the 31 functional species for each specific maturational subset. Significant differences in the frequency of functional subsets is shown between CD45RO^+^ CD27^+^ and CD27^−^CD57^−^ memory CD4^+^ T cells by a * (*P*<0.05) and for differences between CD45RO^+^ CD27^+^ and CD27^−^CD57^+^ memeory CD4^+^ T cells by a † (*P*<0.05).

We have previously shown that in HIV-uninfected individuals as CMV-specific CD4^+^ T cells mature their response patterns change [14]. More mature cells infrequently produced IL-2 yet frequently mobilized CD107a and produced MIP-1β. To determine if a similar phenomenon was observed in PBMC from HIV infected individuals, we assessed the functional properties of CMV-specific CD4^+^ T cells that were at different stages of maturation based upon expression of CD45RO, CD27, and CD57 in the six HIV infected individuals shown in Figure 1. In CMV-specific CD4^+^ T cells polyfunctionality increased with maturation (Figure 3A & B). The median number of different functional responses increased from 2.7 (2.2–3.3) in CD45RO^+^CD27^+^ CD4^+^ T cells, to 3.2 (2.5–3.8) in CD27^−^CD57^−^ CD4^+^ T cells, to 3.7 (2.5–4.1) in CD27^−^CD57^+^ CD4^+^ T cells (***P***<0.05).

Production of IFNγ, TNFα, and MIP-1β and mobilization of CD107a was the most common functional combination among CMV-specific T cells (Figure 1B). The proportion of CMV-specific CD4^+^ T cells expressing this combination of functions increased with maturation, representing a median of 4% of the CD45RO^+^CD27^+^ subset, 24% of the CD27^−^CD57^−^ subset, and 54% of the CD27^−^CD57^+^ subset of responding CD4^+^ T cells (Figure 3B). A similar pattern was seen for CD4^+^ T cells which produced IFNγ, TNFα, and MIP-1β in responses to antigenic stimulation. These data show that CMV-specific CD4^+^ T cells from HIV infected individuals produce MIP-1β just as frequently as CMV specific CD4^+^ T cells from non-HIV infected individuals [14].

### CMV-specific CD4^+^ T cells produce both MIP-1α and MIP-1β

**Figure 4:**
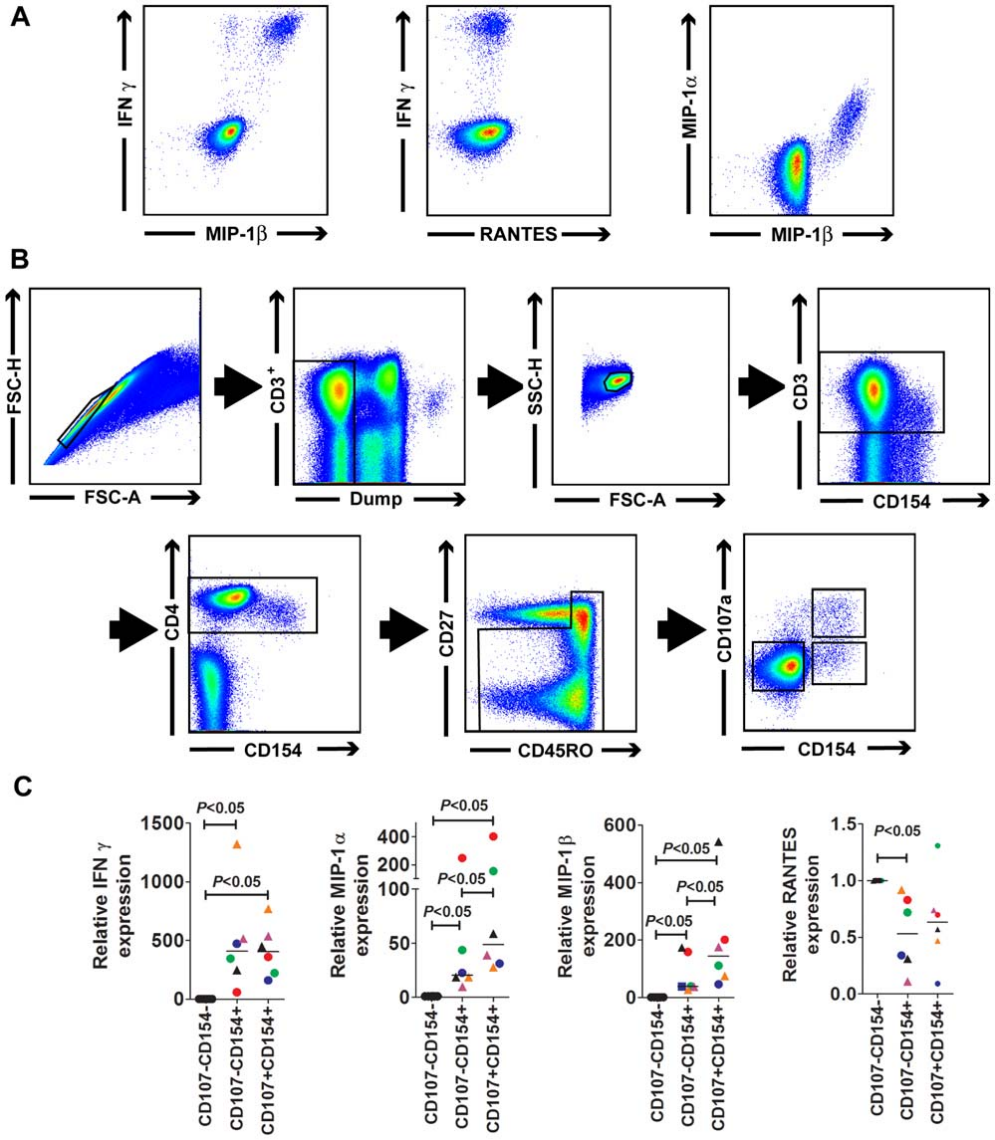
Stimulation of pp65-specific CD4+ T cells increased production of stainable IFNγ, MIP-1α and MIP-1β and their respective mRNA, but not RANTES and its mRNA. A) A plot of CD4+ T cells stimulated with overlapping pp65 15mers shows populations of pp65-specific cells which stain for IFNγ vs MIP-1β, IFNγ vs RANTES and MIP-1α vs. MIP-1β. Although staining for MIP-1α and MIP-1β were clearly visible, no evidence of RANTES staining was apparent. B) PBMC were incubated with overlapping pp65 peptides, anti-CD154 PE, anti-CD107a Alexa680 and monensin for 5 hr. Cells were then stained as described in Materials and Methods, and live CD14^−^CD19^−^CD3^+^CD8^−^CD4^+^ memory cells were sorted into CD107^−^CD154^−^, CD107^−^CD154^+^ and CD107^+^CD154^+^ populations as shown. C) The relative expression of IFNγ mRNA, MIP-1α mRNA, and MIP-1β mRNA were all increased compared to the constitutively expressed glucuronide synthetase mRNA in pp65-specific cells (*P*<0.05). Expression of IFNγ mRNA was no higher in pp65-specific cells which surface mobilized CD107a than in cells that did not surface mobilize CD107a. The ratio of MIP-1α mRNA, and MIP-1β mRNA to hGUS were increased in pp65 specific cells which surface mobilized CD107a compared to those which did not (*P*<0.05). RANTES mRNA was decreased in pp65-specific cells. Results from individuals are color coded. HIV-uninfected individuals are represented with circles; HIV-infected individuals are represented with triangles.

Production of MIP-1β by CMV-specific CD4^+^ T cells may protect these cells from HIV infection. *In vitro* experiments have shown that in addition to MIP-1β the β-chemokines RANTES and MIP-1α can also prevent HIV infection [21],[22],[29],[30]. If the production of β-chemokines is protective against HIV infection *in vivo*, determining which of these chemokines is produced in response to CMV antigens is important. We therefore stimulated PBMC from 3 HIV-infected individuals and 3 HIV-uninfected individuals with pp65 peptides and stained for the production of MIP-1α, MIP-1β, and RANTES (Figure 4A). Median frequency of pp65 specific CD4^+^ T cells was 1.75 (0.7–12.4)%. No evidence of RANTES production was found in these assay. MIP-1α and MIP-1β appeared to stain similarly with a median of 82 (65–97)% of the MIP producing CD4^+^ T cells co-staining for both chemokines. However, the interpretation of these data are complicated by 2 factors. We found that the antibody used to stain MIP-1β cross reacted with the MIP-1α isoform to some extent, making it difficult to determine the exact frequency of cells producing this chemokine (data not shown). It was clear however that staining for MIP-1β identified all MIP-producing species. For this reason MIP-1β was used for all subsequent sorting experiments. Secondly, it is possible that RANTES is stored in CD4^+^ T cells in a form which is not accessible to our antibody staining.

To further assess CMV-induced MIP-1α, MIP-1β and possible RANTES production, we determined relative chemokine-specific mRNA levels using non-cross reacting primers and probes. To identify antigen specific CD4^+^ T cells we stimulated cells with antigen and surfaced stained CD4^+^ T cells for 2 functional responses induced by cognate antigen stimulation; surface mobilization of CD107a and surface expression of CD154, a general marker of CD4^+^ T cell activation expressed on most CMV-specific CD4^+^ T cells [31]. Live memory CD4^+^ T cells were sorted into CD154^−^CD107a^−^, CD154^+^CD107a^−^ and CD154^+^CD107a^+^ populations (Figure 4B) and the relative amounts of IFNγ, MIP-1α, MIP-1β, and RANTES mRNA compared to the amount of mRNA produced by the housekeeping gene human glucuronide synthetase (hGUS). The ratio of the cytokine or chemokine mRNA to hGUS mRNA in antigen specific cells (CD154^+^) was compared to the ratios determined in the cells which did not respond to antigen (CD107a^−^CD154^−^). These ratios were used to assess the relative amounts of IFNγ, MIP-1α, MIP-1β and RANTES mRNAs (Figure 4C). Median ratios for IFNγ∶hGUS, MIP-1α∶hGUS , and MIP-1β∶hGUS were significantly higher in pp65-specific cells than in cells that were not stimulated by pp65 (***P***<0.05). Unlike IFNγ, both MIP-1α and MIP-1β median chemokine∶hGUS mRNA ratios were also higher in antigen specific CD4^+^ T cells (CD154^+^) that expressed CD107a than in those that did not (***P***<0.05). The increase in MIP production in cells that are CD107^+^ compared to cells that are CD107^−^ is consistent with the increased frequency of MIP producing cells in the CD107a^+^ population shown in Figure 1B. No increase in production of RANTES mRNA was observed in pp65 specific CD4+ T cells stimulated with antigen. None-the-less, the presence of measurable RANTES RNA suggested that RANTES may be produced in CD4^+^ T cells even in the absence of antigenic stimulation. To assess this we measured the production of RANTES in unstimulated memory CD4^+^ T cells. We could not consistently measure RANTES in 6 h incubations, but we could measure RANTES in 24 h incubations containing 0.7µg monensin/ml (Figure S1). In much the same way that the frequency of MIP-1β producing cells were greater in more mature cells, we found that the frequency of RANTES producing cells were greater in CD27^−^CD57^+^ memory CD4^+^ T cells than in CD27^−^CD57^−^ memory CD4^+^ T cells (***P***<0.05) which were in turn were greater than in CD27^+^ memory CD4^+^ T cells (***P***<0.05). In matched incubation, no RANTES was found in cells which produced IFNγ in response to stimulation with SEB. RANTES was still found in memory CD4^+^ T cells which did not produce IFNγ but the frequency of RANTES producing cells was less than was found in incubations that did not contain SEB. These data suggest that RANTES is produced in more mature memory CD4+ T cells and then released after stimulation.

### Surface expression of CCR5 was lower in MIP-1β producing than non- MIP-1β producing CMV-specific CD4^+^ T cells

**Figure 5:**
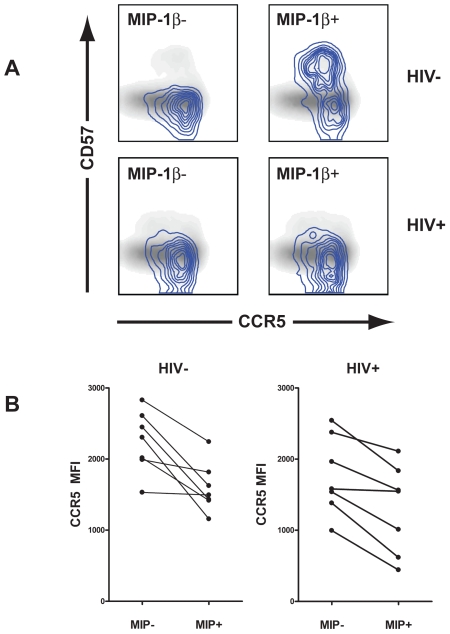
MIP-1β producing pp65-specific CD4^+^ T cells have lower surface expression of CCR5. A) pp65-specific CD4^+^ T cells which either produce or do not produce MIP-1β are overlaid onto a 2 dimensional histograph of memory CD4^+^ T cells plotted against CD57 and CCR5 surface expression for a representative HIV-uninfected and HIV-infected individuals. MIP-1β^+^ and MIP-1β^−^ memory CD4^+^ T cells, shown by blue isobars, are overlayed on 2 dimensional density plots showing expression of CCR5 and CD57. B) For all seven of the non-HIV and all seven of the HIV infected individuals, pp65 specific CD4^+^ T cells which produced MIP-1β had lower MFIs for CCR5 surface expression than cells which did not produce MIP-1β (*P*<0.05).

In addition to MIP-1α and MIP-1β preventing binding of gp120 to CCR5 by steric hindrance, MIP-1α and MIP-1β can also down-regulate surface expression of CCR5 [20],[32]. To see if surface expression of CCR5 was down-regulated in MIP-1α and MIP-1β producing CD4^+^ T cells, we compared the frequency of surface expression of CCR5 on CMV-specific CD4^+^ T cells that produced MIP-1β (which also contained all MIP-1α producing cells) to that observed on cells which did not produce MIP-1β. We used production of IFNγ as a marker of CMV-specific cells. CD4^+^ T cells which responded to CMV were divided into MIP-1β producing and non-MIP-1β producing cells (Figure 5A). MIP-1β producing CD4^+^ T cells had significantly lower CCR5 surface expression than non-MIP-1β producing cells (***P***<0.05). Although CD57^−^ CD4^+^ T cells could be divided into 2 groups of cell, CCR5^+^ and CCR5^−^ cells, expression of CCR5 on CD57^+^ cells varied from individual to individual, with some CD57^+^ cells being predominantly CCR5^+^, some being CCR5^−^ and some showing intermediate levels of CCR5 surface expression. As a result of this variability we also compared mean fluorescence of CCR5 staining between CMV-specific cells which expressed MIP-1β and cells which did not (Figure 5B). Again, in both HIV infected individuals and non-HIV infected individuals, CMV-specific cells which expressed MIP-1β showed lower CCR5 MFIs than cells which did not express MIP-1β (***P***<0.05). To determine if this decrease in surface CCR5 staining was caused by *in vitro* MIP-1α or MIP-1β production in Brefelden A containing assays we added pre-titered amounts of purified anti-MIP-1α and MIP-1β to Brefelden A containing assays in which the total pp65 induced MIP-1β was 1.35 (0.7–11.5)% of the total CD4^+^ population. No increase in CCR5 expression was observed with addition of anti-MIP-1α and MIP-1β blocking antibodies in either CD57^−^ or CD57^+^ memory CD4^+^ T cells (data not shown). Reversal of MIP-1α and MIP-1β induced CCR5 down regulation was used to titer anti-MIP-1α and MIP-1β antibody (Figure S2).

### CMV-specific CD4^+^ T cells that produce MIP are less frequently infected with HIV than those that do not

**Figure 6:**
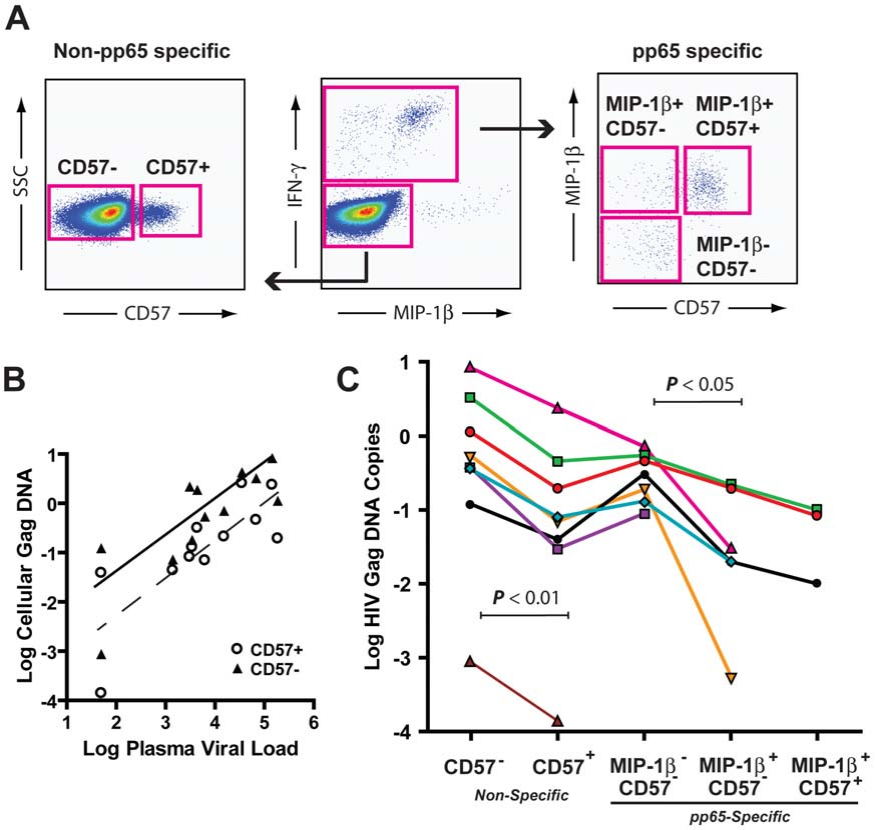
MIP-1β producing pp65-specific CD4^+^ T cells have a lower frequency of HIV infection than do non-MIP-1β producing pp65 specific CD4^+^ T cells. A) PBMC were incubated in the presence of BFA and pp65 15mers overlapping by 11 for six hours. pp65-specific CD4^+^ T cells were separated from memory CD4^+^ T cells based on production of IFNγ. Cells which were non-pp65 specific were sorted into CD57^+^ and CD57^−^ populations. Cells which were pp65-specific were sorted in MIP-1β^+^CD57^+^, MIP-1β^+^ CD57^−^, or MIP-1β^−^CD57^−^ populations. Quantitative real time PCR was done on each of these populations to determine the frequency of cell associated gag DNA. B) In all 12 individuals studied, CD57^+^ non-pp65 specific cells (open circles) had lower frequencies of cell associated gag DNA than did CD57^−^ non-pp65-specific cells (solid triangles) (*P*<0.01). For both CD57^+^ and CD57^−^ CD4^+^ T cells log plasma viral load was linearly related to log cell associated gag DNA (*P*<0.01). R^2^ values were 0.62 for CD57^+^ cells and 0.67 for CD57^−^ cells. C) A plot of the log of cell-associated gag DNA in pp65-specific and non-specific CD4^+^ T cells from 8 HIV-infected individuals. Non-pp65-specific CD4^+^ T cells that were CD57^+^ had lower amount of cell associated Gag DNA than did non-pp65 CD57^−^ CD4^+^ T cells (*P*<0.01). CD57^−^ CD4^+^ T cells which were pp65-specific and produced MIP-1β had lower amounts of cell associated Gag DNA than did similar pp65-specific CD4^+^ T cells that did not produce MIP-1β (*P*<0.05). Where values are not shown, the amount of cell associated gag DNA was below the detection limit of the method.

To address whether MIP-1α and MIP-1β production are protective *in vivo* we used cell associated gag DNA as a measure of the HIV infection history of CD4^+^ T cells [10] in CMV-specific CD4^+^ T cells which did, or did not, stain for MIP-1β after antigenic stimulation. MIP-1β producing CD4^+^ T cells occur more frequently in CD57^+^ cells than in CD57^−^ memory CD4^+^ T cells [14]. The frequency of HIV infection of CD57^+^ memory CD4^+^ T cells has been reported to be approximately 10 fold less than in CD57^−^ memory CD4^+^ T cells [33]. To ensure that the rate of HIV infection of CD57^+^ memory CD4^+^ T cells did not interfere with the interpretation of the effect of MIP-1β production on HIV infection, we also sorted CMV-specific memory CD4^+^ T cells (cells that were CD45RO^+^ or CD45RO^−^CD27^−^) into populations which did or did not respond to CMV pp65 peptides based on IFNγ production after a six hour incubation. CMV-specific CD4^+^ T cells were then sorted into 3 populations: MIP-1β producing CD4^+^ T cells that were CD57^+^, MIP-1β producing CD4^+^ T cells that were CD57^−^, and CD4^+^ T cells that did not produce MIP-1β and were CD57^−^ (Figure 6A). There were too few antigen-specific CD57^+^ cells that did not produce MIP-1β to sort. Cells which did not respond to pp65 were sorted into CD57^+^ and CD57^−^ populations. In all 5 populations the amount of cell associated gag DNA was determined and then normalized to a per cell amount based on the quantity of albumin-encoding DNA.

For each individual studied the amount of cell-associated HIV gag DNA was less in the unstimulated population that was CD57^+^ than in the unstimulated CD57^−^ population. The median cell-associated gag DNA in the CD57^+^ population was 4 (1.3–26.6) fold less than in the CD57^−^ population (Figure 6B). Plasma viral load correlated with cell-associated gag DNA in both the CD57^+^ (***P***<0.01, R^2^ = 0.62) and the CD57^−^ populations (***P***<0.01, R^2^ = 0.67) based on linear regression analysis (Figure 6B).

Within CD57^−^ CMV-specific CD4^+^ T cells, cell associated gag DNA was 11 (2.5–333) fold lower in CD4^+^ T cells which produced MIP-1β than in those that did not (***P***<0.05). While we did not have adequate numbers of cells to quantify gag DNA from all donors in all populations, there was a consistent pattern that among MIP-1β producing cells, expression of CD57 was associated with even lower frequencies of HIV gag DNA (Figure 6C).

We also stimulated PBMC from HIV infected individuals with SEB to determine if CD57^−^ memory CD4^+^ T cells producing IFNγ and MIP-1β had lower levels of infection than those that produced IFNγ, but no MIP-1β. We did not find a significant difference between these two populations (Figure S3).

## Discussion

In this study we compared the maturation and function of CMV- and HIV-specific CD4^+^ T cells in individuals with similar frequencies of CMV- and HIV-specific CD4^+^ T cells. We show the following: i) consistent with prior reports, CMV-specific CD4^+^ T cells are more frequently CD27^−^ and CD57^+^ than are HIV-specific CD4^+^ T cells; ii) CMV-specific CD4^+^ T cells are more polyfunctional in that they more frequently mobilize CD107a and produce MIP-1β than do HIV-specific CD4^+^ T cells; iii) CMV specific CD4+ T cells produce two β-chemokines in response to antigenic stimulation, MIP-1α and MIP-1β; and iv) CMV-specific CD4^+^ T cells that express MIP-1β and are either CD57^+^ or CD57^−^ are less frequently infected with HIV *in vivo* than CMV-specific CD4^+^ T cells which are CD57^−^ and do not express MIP-1β.

Our data, which show decreased HIV infection of MIP-1β producing CMV-specific CD4^+^ T cells compared to similar CMV-specific CD4^+^ T cells which do not produce MIP-1β, support a large body of *in vitro* data that show protection of CD4^+^ T cells from HIV infection by β-chemokines. Although we only showed a decreased rate of infection of CMV-specific CD4^+^ T cells with respect to MIP-1β, this finding almost certainly extends to MIP-1α also. Our data show that most CMV-specific CD4^+^ T cells that produce MIP-1β in response to antigenic stimulation concurrently produce MIP-1α. Both chemokines down-regulate surface expression of CCR5 in CD4^+^ T cells *in vitro*. Both chemokines are protective *in vitro*. To our knowledge these data are the first to suggest that autocrine production of MIP-1α and MIP-1β by antigen-specific CD4^+^ T cells is protective against HIV-infection *in vivo*. We were unable to show a difference in the HIV infection history of CD57^−^ memory CD4^+^ T cells which produced MIP-1β and IFNγ and those which produced IFNγ when stimulated with the superantigen SEB. The significance of this finding is unclear. Whereas pp65 stimulates cells with similar cytokine profiles, maturation levels, and histories of exposure to cognate antigen, SEB stimulates cells with multiple specificities, maturation levels, and histories of exposure to cognate antigen. This could affect their *in vivo* HIV exposure histories. For instance, SEB responsive cells that produce MIP-1β could have relatively high levels of HIV DNA if those cells were CMV-specific and had responded to CMV when the subject was co-infected with HIV, while a MIP-1β non-producing cell could have virtually no history of HIV exposure if its specificity was to a pathogen which the subject had not encountered during the time of HIV infection (ie. a measles-specific cell).

The mechanism of protection of CMV-specific CD4^+^ T cells *in vivo* is probably similar to that proposed for the protection of CD4^+^ T cells *in vitro*. The entry of R5-tropic HIV is blocked by MIP-1α and MIP-1β and production of these chemokines by CMV-specific CD4^+^ T cells results in a concentration gradient with the highest concentrations of MIP-1α and MIP-1β near the cells which produce them. This concentration gradient should result in more frequent occupancy of the HIV CCR5 binding site, and lower densities of CCR5 on the cell surface because of MIP-1α and MIP-1β induced down-regulation of surface CCR5 expression [34] than on similar cells which do not produce MIP-1α and MIP-1β. The decrease in CCR5 that we show in Figure 5 most likely is an underestimation of the effects of MIP production on CCR5 expression. Brefeldin A which traps nascent proteins in the endoplasmic recticulum was used in almost all of the experiments reported in this manuscript and most likely severly decreased MIP secretion in these assays. This supposition is supported by data showing down regulation of CCR5 expression on memory CD4^+^ T cells stimulated by anti-CD2, CD3 CD8 activation beads in the absence of Brefeldin A; the reversal of this affect by anti-MIP-1α and MIP-1β; and the failure of anti-MIP-1α and MIP-1β to increase CCR5 expression on activated cells in the presence of Brefelden A. It therefore seems likely that the affect of MIP-1α and MIP-1β produced during the course of BFA containing incubations on CCR5 expression was minimized. The role of RANTES production in the downregulation of CCR5 and the protection of CD4^+^ T cells is unclear. Our data does not show an increase in RANTES mRNA production with short term stimulation but does show constitutive synthesis of RANTES in unactivated memory CD4^+^ T cells in short term *ex vivo* culture, particularly in cell which are either CD27^−^ or CD27^−^CD57^+^. The absence of RANTES staining in SEB stimulated cells which produce IFNγ suggest that RANTES is released from CD27^−^ and CD27^−^CD57^+^ memory CD4^+^ T cells when they are activated.

CMV-specific CD4^+^ T cell responses are highly polyfunctional and have decreased surface expression of CD27 and variable surface expression of CD57 (Figure 3B). In contrast, HIV-specific CD4^+^ T cells had high levels of surface expression of CD27 and relatively low levels of CD57 expression compared to CMV-specific CD4^+^ T cells. Although the frequency of IFNγ, TNFα and IL-2 production were similar in both sets of cells, most HIV-specific cells rarely showed evidence of degranulation or MIP-1β production. In the individuals we studied there was an exception to this generalization. In the subject with the highest frequency of gag-specific CD4^+^ T cells, those cells were functionally and maturationally reminiscent of CMV-specific CD4^+^ T cells. They had lower surface expression of CD27 (11%) and greater surface expression of CD57 (50%). This individual also had the highest frequency of CD107a surface mobilization (29%) and MIP-1β production (61%) in response to stimulation with HIV gag peptides of the six individuals we studied. The HIV-specific response we observed in this individual was due to CD4^+^ T cell clones that recognized 3 specific peptide epitopes: p17_31–46_, YKLKHIVWASRELER; p24_18–33_, PRTLNAWVKVVEEKA; and p24_133–148_, WIILGLNKIVRMYSP (data not shown). All three peptides were of similar response frequency and corresponded with regions previously reported as class II epitopes [35]. These data show that more mature HIV-specific CD4^+^ T cells producing higher amounts of IFNγ, TNFα and MIP-1β can be generated and maintained in HIV infection.

These studies demonstrate that not only do CMV-specific CD4^+^ T cells differ in their maturational and functional profile from HIV-specific CD4^+^ T cells, but those specific functions are associated with protection against infection *in vivo*. Specifically, autocrine production of the chemokines MIP-1α and MIP-1β appears to be the predominant mechanism involved in protection against *in vivo* infection of CMV-specific CD4^+^ T cells. Induction of a functional profile in HIV-specific CD4^+^ T cells similar to that seen in CMV-specific CD4^+^ T cells could result in a more effective and durable immune response during HIV infection. Our data showing decreased rates of HIV infection in MIP-1β producing CD4^+^ T cells and the data of others showing increased cytokine production in more polyfunctional antigen-specific CD4^+^ T cells [36] suggest that inducing polyfunctional CD4^+^ T cells which produce MIP-1α and MIP-1β could be important for both therapeutic and preventative HIV vaccines. While the identification of a functional profile within CD4^+^ T cells that is relatively protective against HIV infection *in vivo* is enlightening, the ultimate goal is to find ways of inducing such polyfunctional CD4^+^ T cells through vaccination, potentially providing the immune system with cells that had strong effector and helper functions against HIV, and were simultaneously relatively protected against infection and deletion by HIV. Recent discoveries into how to induce polyfunctional CD4^+^ T cells through vaccination offer hope that the practical application of these findings is on the near horizon [36]–[39].

## Materials and Methods

### Ethics statement

This study was conducted according to the principles expressed in the Declaration of Helsinki. This study was approved by the Institution Review Board of the NIAID and the Whitman Walker Clinic. All patients provided written informed consent for the collection of samples and subsequent analysis.

### Subjects

Peripheral blood mononuclear cells (PBMC) were obtained from twelve HIV uninfected, CMV seropositive individuals participating in the NIH research apheresis program. PBMC were obtained from eighteen HIV infected individuals either recruited from the Whitman Walker Clinic of Washington D.C. or through the Vaccine Research Center apheresis protocol. Informed consent was obtained from all subjects prior to enrollment into this study (Table 1). In some cases the requirement for specific experiments determined the patients used. For the comparisions of gag-specific CD4^+^ T cell responses to pp65 specific CD4^+^ T cell responses we used PBMC from HIV-infected individuals with gag- and pp65-specific CD4^+^ T cell responses which were greater than 0.1% of the total CD4^+^ population. All of the individuals which we identified for this cohort had viral loads of <1000 (Figure 1–3). For experiments where only pp65 specific CD4^+^ T cells were studied we tried to represent individuals with a broader range of viral load. Finally large numbers of cells were required for the determination of cell-associated Gag DNA content in CD57^−^ IFNγ^+^MIP-1β^+^ and CD57^−^ IFNγ^+^MIP-1β^−^ CD4^+^ T cells. For these experiments we only used samples for which apheresis samples were available (Figure 6). All experiments were done using cryopreserved PBMC. PBMC were prepared within 2 hours of apheresis or blood draw. Plasma HIV load and CD4^+^ T cells counts were determined by a CLIA certified laboratory.

### Cell stimulations

Frozen PBMC were thawed, washed twice with RPMI 1640 supplemented with 10% heat inactivated fetal calf serum, 100 U/ml penicillin G, 100U/ml streptomycin sulfate, and 1.7mm sodium glutamine (R-10). Cells were then re-suspended in R-10 containing 10 U/ml DNase I (Roche Diagnostics) and rested for two hours before being washed with R-10 and used for experiments. All experiments characterizing the immune response of antigen-specific CD4^+^ T cells were done at 2×10^6^ PBMC/ml in the presence of 1 µg/ml each of αCD28 and αCD49d (BD Bioscience), and10µg/ml brefeldin A (BFA) (Sigma Chemical Company) in the absence or presence of peptide antigens unless otherwise noted. Cell stimulation in which surface mobilization of CD107a were measured were done as above, but in addition a pre-titered amount of αCD107a and 0.7 µg monensin/ml (BD Bioscience) was added [40]. For determination of RANTES production, only monensin was addeded. Incubations were 6 h in duration unless otherwise noted. Since we could only identify maturational phenotypes for antigen-specific responses by cytokine or chemokine production all maturational data represent the maturation state of these cells after a 6h incubation period.

Incubations used for determination of mRNA levels were done in a different manner. Cells were thawed and rested as described above, but cell incubations were done in 2 ml volumes of R-10 containing 5×10^6^ PBMC/ml. Incubations contained 1µg/ml αCD28, 1 µg/ml αCD49d and peptide antigens. After a 1h pre-incubation period pre-titered amount of CD107a-Alexa680 and CD154-PE along with 0.7µg monensin/ml were added [31]. Cells were then incubated for an additional 5 hours before staining.

Anti-CD2, CD3, CD28 activation beads were obtained from Miltenyi Biotec and were prepared as described by the manufacturer. Cells were activated by the addition of 0.5 beads/cell and incubated for 5 h before staining.

### Antigens

One hundred and twenty-two 15mers overlapping by 11 amino acids corresponding to the entire clade B HXBc2/Bal R5 chimeric HIV gag protein sequence were pooled and dissolved in DMSO. Gag peptides were obtained from Boston Bioscience and were greater than 70% pure. One hundred and thirty-eight 15mers overlapping by 11 amino acids corresponding to the entire pp65 protein sequence were pooled and dissolved in DMSO. CMV pp65 peptides were obtained from JPT peptide technology and were >70% pure. In pooled peptide mixes, each peptide was at a concentration of 400 µg/ml. Five µl were added for each ml of assay volume. Final concentration of peptides was 2 µg/ml.

### Antibodies

Directly conjugated monoclonal antibodies (mAbs) specific for the molecules listed were obtained from the following: IL-2-allophycocyanin (APC), CD3-Cy7APC, IFNγ-FITC, TNFα-Cy7PE, MIP-1β-PE, CD154-PE, RANTES-PE and CCR5- Cy7PE were from BD Biosciences; CD45RO-TRPE, CD27-Cy5PE were from Beckman Coulter; CD4-Cy55PE was from Caltag; MIP-1α-APC was from R&D. The following antibodies were conjugated in our laboratory according to standard protocols (http://drmr.com/abcon/index.html): CD107a-Alexa680, CD8-QD705, CD57-QD565, CD14-Pacific Blue and CD19-Pacific Blue.

### Immunofluorescence staining

Stimulated PBMC used for intracellular cytokine staining were washed and pre-stained for 10 min with a pre-titered amount of LIVE/DEAD fixable violet dead cell stain (Molecular Probes). In some incubations cells were also pre-stained with an anti-CCR5 antibody. After preliminary staining, cells were then surface stained with a mixture of pre-titered amounts of directly conjugated antibodies to CD27, CD45RO, CD57, CD8, CD19, and CD14 made to a total volume 100µl with Delbecco's phosphate buffered saline (PBS). Cells were stained for 30 min at room temperature in the dark. Cells were then washed and permeabilized using the cytofix/cytoperm kit (BD Biosciences) according to the manufacturer's instructions. After intracellular staining for CD3, CD4, IFNγ, MIP-1α, MIP-1β, TNFα, RANTES and/or IL-2 cells were washed one final time and fixed in PBS containing 1% paraformaldehyde and then stored in a 4°C refrigerator. Flow cytometric analysis was done within 24 h of staining.

Stimulated cells used for mRNA quantitation were pre-stained for 10 min with LIVE/DEAD fixable violet dead cell stain (Invitrogen) and then stained with pre-titered amounts of directly conjugated antibodies to CD27, CD45RO, CD19, CD14, CD3, CD8 and CD4 and made to a total volume 100µl with Dulbecco's phosphate buffered saline (PBS). Cells were stained for 30 min at room temperature in the dark, washed once and then flow cytometric sorting immediately done.

### Flow cytometric analysis

Cells were analyzed with a modified LSRII (BD Immunocytometry Systems) equipped for the detection of 18 fluorescence parameters. For all 12 color flow analysis between 500,000 and 1,000,000 events were collected for each sample. Electronic compensation was conducted with antibody capture beads (BD Biosciences) stained separately with individual mAbs used in test samples. All analytical gating was was performed using FlowJo version 8.2 (Tree Star) as described previously [14]. Although there were variations in gating based on the population of interest all CD4^+^ T cells were identified in the same manner. Singlet cells were sorted base on Forward Scatter Height (FSC-H) and Forward Scatter Area (FSC-A). Dead cells, B cells, and monocytes were excluded by staining with either LIVE/DEAD fixable violet dead cell stain or CD14 and CD19 staining. CD3^+^,CD8^−^ and CD3^+^CD4^+^ cells were sequentially selected. Identification of CD107a, IFNγ, IL-2 MIP-1β and TNFα was done in either CD3^+^CD8^−^CD4^+^ T cells, or CD3^+^CD8^−^CD4^+^ T cells that were CD27^+^CD45RO^+^, CD27^−^ 57^−^ or CD27^−^CD57^−^ cell populations. CCR5 gating was done on memory CD3^+^CD8^−^CD4^+^ T cells that either produced, or did not produce MIP-1β. Memory cells were defined as all CD3^+^CD8^−^CD4^+^ cells that were not CD27^+^CD45RO^−^. Graphs were made using Pestle Version 1.5 (provided by M. Roederer, NIH, Bethesda, MD) and Spice Version 4.1 (provided by M. Roederer, NIH, Bethesda, MD).

### Cell sorting

Cell sorting for quantitation of cell-associated Gag-DNA was accomplished using a FACS Aria cell sorter (BDIS) at 70 lb/in^2^. CD19 Cascade Blue, CD14 Cascade Blue, CD45RO TRPE, CD27 Cy5PE, CD57 QD565, CD8 QD705, CD3 Cy7APC, CD4 Cy55PE, IFNγ FITC, MIP-1β and LIVE/DEAD fixable violet dead cell stain were used to stain cells. Typically at least 50 million PBMC were sorted for each experiment in which cell associated gag DNA was determined.

Sorted populations were consistently >98% pure. Immediately upon completion of cell sorting cells were centrifuged in 1.5 ml polypropylene conical test tubes, the supernatant removed and 25–100 µl of 10 mM TRIS buffer containing proteinase K added. Cells were then incubated at 56°C for 1 hour, and then at 90°C for 10 min. Cell debris was removed by centrifuged and samples stored at −80°C until quantitative real time PCR (qPCR) was performed.

Cell sorting for quantitation of mRNA was accomplished using a FACS Aria cell sorter (BDIS) at 70 lb/in^2^ (Figure 4B). Cells were sorted in the following manner: Single cells were sorted based on Forward Scatter Height and Forward Scatter Area. Dead cells, B cells, and monocytes were excluded by staining with either LIVE/DEAD fixable violet dead cell stain or CD14 and CD19 staining. CD3^+^ and CD4^+^ cells were sequentially selected, then memory CD4^+^ T cells were selected based on CD27 and CD45RO surface staining. Live antigen-specific memory CD4^+^ T cells were sorted based on surface expression of CD154 and CD107.

### qPCR

Quantification of HIV gag DNA in sorted CD4^+^ T cells was performed by quantitative PCR (qPCR) by means of a 5′ nuclease (TaqMan) assay with an ABI7700 system (Perkin Elmer, Norwalk, CT) as previously described [33],[41]. Standards were constructed for absolute quantification of gag and albumin copy number and were validated with sequential dilution of 8E5 cell lysates that contain one copy of gag per cell. Duplicate reactions were run and template copies calculated using ABI7700 software.

### Quantitation of MIP-1α, MIP-1β, IFNγ RANTES and hGUS mRNA

Memory T cell subsets were sorted directly into RNALater (Ambion) and centrifuged at 10,000g for 5 min. Supernatents were discarded and total RNA extracted using RNAse aqueous for PCR (Ambion). mRNA was then purified using the Oligotex mRNA extraction kits per the manufactures instructions (Qiagen).

Purified mRNA was added directly to the iScript one step quantitative RT PCR reaction (Bio-RAD) containing either IFNγ, MIP-1α, MIP-1β, RANTES primers and probes or the normalizing primers and probes for human β-glucuronidase (hGUS). We used the following oligonucleotide sequences: MIP-1α forward primer, GACTACTTTGAGACGAGCAGCCA; MIP-1α reverse primer, GCCGGCTTCGCTTGGT, MIP-1α probe, FAM- TGCTCCAAGCCCGGTGTCATCTTC-BHQ1 *(Farber,J J.M. personal communication)*; MIP-1β forward primer, AGCGCTCTCAGCACCAATG; MIP-1β reverse primer, TTCCTCGCGGTGTAAGAAAAG; MIP-1β probe FAM-CTCAGACCCTCCCACCGCCTGC-BHQ1 *(Farber,J J.M. personal communication)*; RANTES forward primer, ACCAGTGGCAAGTGCTCCA; RANTES reverse primer, GCACACACTTGGCGGTTCT; RANTES probe, FAM- CCAGCAGTCGTCTTTGTCACCCGA-BHQ1 *(Farber,J J.M. personal communication)*; IFNγ forward primer, CGAGATGACTTC GAAAAGCTGAC; IFNγ reverse primer, GGCGACAGTTCAGCCATCA; IFNγ probe, FAM-TTGAATGTCCAACGCAAAGCAATACATGA-BHQ1 *(Horner, R. M., personal communication)*; hGUS forward primer, CTCATTTGGAATTTTGCCGATT, hGUS reverse primer, CCGAGTGAAGATCCCCTTTTTA; and hGUS probe, TGAACAGTCACCGACGAGAGTGCTGG. Expression levels of human IFNγ, MIP-1α, MIP-1β, and RANTES were normalized to hGUS and calculated based on the ΔΔCT method [42].

### Statistics

Comparison between groups was performed using a criterion of significance of ***P***≤0.05. All statistical tests were conducted using SPSS for Windows (SPSS Inc., Chicago, IL) or JMP (SAS, Cary, NC). Unless specifically noted all values given are medians (range). Pair-wise comparisons were made using a Wilcoxon signed rank statistic.

## Supporting Information

Figure S1 Production of RANTES by CD4^+^ T cells increases with maturation. PBMC from an HIV-uninfected individuals were incubated for 24 h with and without the presence of 1µg SEB/ml. All incubations contained 0.7µg monensin/ml. Cells were surface stained for CD27, CD45RO, CD57, CD14 and CD19 and a live dead cell dye as described in Materials and Methods. Cells were then permeabilzed, washed and stained for CD3, CD4, CD8, IFNγ and RANTES. A.) Although we could not routinely identify RANTES producing cells in unstimulated cells in 6 h incubations, we could do so in 24 h incubations. Addition of SEB resulted in a decrease in the amount of RANTES identified in these assays, No RANTES was found in IFNγ producing cells. Numbers in the bottom portion of the graph represent the percentage of cells which did, and did not produce RANTES. B.) RANTES production increased with maturational phenotype with CD27^+^ CD4^+^ memory cells showing the lowest frequency of RANTES production, CD27^−^CD57^−^ CD4^+^ memory cells showing an intermediate level, and CD27^−^CD57^+^ cells showing the highest frequency of RANTES producing cells. C.) In a cohort of six HIV-uninfected individuals the difference in frequency of RANTES producing cells between CD27^+^ and CD27^−^CD57^−^ memory CD4^+^ T cells and between CD27^−^CD57^−^ and CD27^−^ and CD57^+^ memory CD4^+^ T cells was significantly different at a level of P<0.05 as determined by a Wilcoxon sign ranked test.
(0.77 MB TIF)

Figure S2 Stimulation of memory CD4^+^ T cells by CD2^+^, CD3^+^, CD28^+^ beads results in preferential stimulation of CD57^+^ CD4^+^ T cells and MIP induced down regulation of surface CCR5 expression. PBMC from an HIV-uninfected individuals were stimulated with 0.5 CD2^+^, CD3^+^, CD28^+^ beads/PBMC for 5 hours. PBMC used to characterize the production of IFNγ and MIP-1β were incubated with BFA; those used to characterize surface expression of CCR5 were not. A.) CD57^−^ memory CD4^+^ T cells were less activated as judged by expression of IFNγ than were CD57^+^ memory CD4^+^ T cells. They also produced less MIP-1β. B.) In CD57^−^ memory CD4^+^ T cells stimulated with CD2^+^, CD3^+^, CD28^+^ beads in the absence of BFA surface expression of CCR5 was decreased compared to that observed in cells that were not stimulated. C.) Similarly, in CD57^+^ memory CD4 T cells stimutated with CD2^+^, CD3^+^, CD28^+^ beads in the absence of BFA surface expression of CCR5 was decreased compared to that observed in cells that were not stimulated. CCR5 down regulation was more marked in CD57^+^ memory CD4^+^ T cells than in non-CD57^−^ memory CD4^+^ T cells. D.) PBMC were stimulated with CD2^+^, CD3^+^, CD28^+^ beads in the absence of BFA with either no anti-MIP-1α or anti-MIP-1β blocking antibody or 2, 5 or 10 µg of anti-MIP-1α and anti-MIP-1β/ml. At the conclusion of 5 h incubations containing CD2^+^, CD3^+^, CD28^+^ beads and no BFA the frequency of surface expression of CCR5 was greater than in incubations containing anti-MIP-1α and anti-MIP-1β blocking antibodies than in matched incubations not containing blocking antibodies. CCR5 surface expression for both CD57^+^ and CD57^−^ memory CD4^+^ T cells are shown as a function of the amount of blocking antibodies in each incubation. The solid line and dashed line represent CCR5 expression of unstimulated CD57^−^ and CD57^+^ memory CD4^+^ T cells, respectively.
(0.67 MB TIF)

Figure S3 SEB stimulated CD57^−^ memory CD4^+^ T cells that produce MIP-1β do not contain lower amounts of cell associated Gag DNA than do no non-MIP-1β producing cells CD57^−^ memory CD4^+^ T cells. PBMC at a concentration of 3E06 PBMC/ml were incubated in the presence of BFA and 1µg/ml SEB. At the end of a 6h incubation period cells were stained and sorted in the same manner as shown in Figure 6. Unlike CMV-specific CD4^+^ T, no significant difference was observed in cell associated Gag DNA in IFNγ producing CD57^−^ cells which produced MIP-1β and those that did not.
(0.10 MB TIF)
