# Supplementary figures and images for: Autocrine Production of β-Chemokines Protects CMV-Specific CD4+ T Cells from HIV Infection
Source: PLoS Pathog. 2009 Oct 30;5(10):e1000646. doi: 10.1371/journal.ppat.1000646 (PMC2763204; doi:10.1371/journal.ppat.1000646)

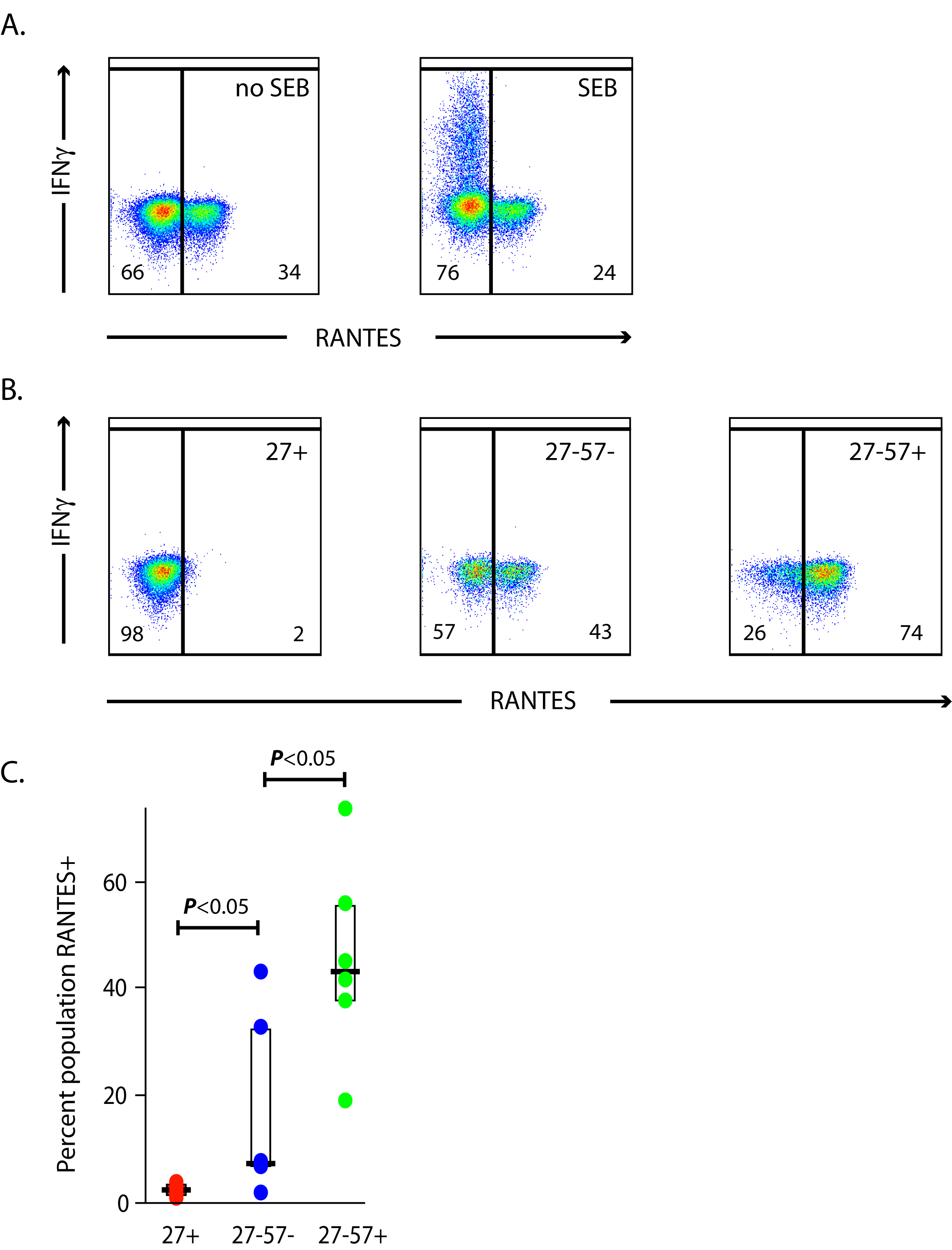

Supplement: Figure S1 — Production of RANTES by CD4+ T cells increases with maturation. PBMC from an HIV-uninfected individuals were incubated for 24 h with and without the presence of 1µg SEB/ml. All incubations contained 0.7µg monensin/ml. Cells were surface stained for CD27, CD45RO, CD57, CD14 and CD19 and a live dead cell dye as described in Materials and Methods. Cells were then permeabilzed, washed and stained for CD3, CD4, CD8, IFNγ and RANTES. A.) Although we could not routinely identify RANTES producing cells in unstimulated cells in 6 h incubations, we could do so in 24 h incubations. Addition of SEB resulted in a decrease in the amount of RANTES identified in these assays, No RANTES was found in IFNγ producing cells. Numbers in the bottom portion of the graph represent the percentage of cells which did, and did not produce RANTES. B.) RANTES production increased with maturational phenotype with CD27+ CD4+ memory cells showing the lowest frequency of RANTES production, CD27−CD57− CD4+ memory cells showing an intermediate level, and CD27−CD57+ cells showing the highest frequency of RANTES producing cells. C.) In a cohort of six HIV-uninfected individuals the difference in frequency of RANTES producing cells between CD27+ and CD27−CD57− memory CD4+ T cells and between CD27−CD57− and CD27− and CD57+ memory CD4+ T cells was significantly different at a level of P<0.05 as determined by a Wilcoxon sign ranked test. (0.77 MB TIF) [file ppat.1000646.s001.tif]

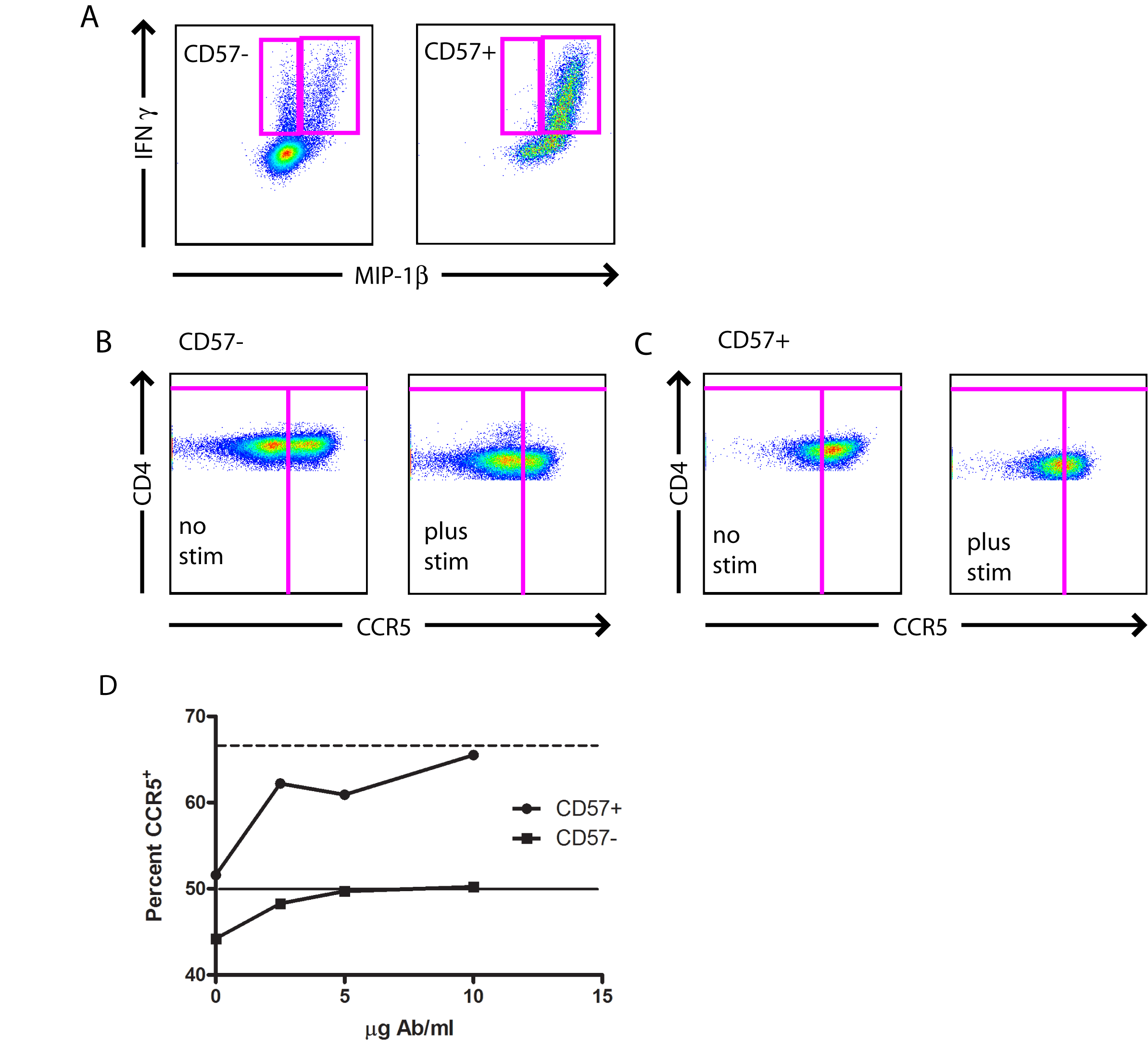

Supplement: Figure S2 — Stimulation of memory CD4+ T cells by CD2+, CD3+, CD28+ beads results in preferential stimulation of CD57+ CD4+ T cells and MIP induced down regulation of surface CCR5 expression. PBMC from an HIV-uninfected individuals were stimulated with 0.5 CD2+, CD3+, CD28+ beads/PBMC for 5 hours. PBMC used to characterize the production of IFNγ and MIP-1β were incubated with BFA; those used to characterize surface expression of CCR5 were not. A.) CD57− memory CD4+ T cells were less activated as judged by expression of IFNγ than were CD57+ memory CD4+ T cells. They also produced less MIP-1β. B.) In CD57− memory CD4+ T cells stimulated with CD2+, CD3+, CD28+ beads in the absence of BFA surface expression of CCR5 was decreased compared to that observed in cells that were not stimulated. C.) Similarly, in CD57+ memory CD4 T cells stimutated with CD2+, CD3+, CD28+ beads in the absence of BFA surface expression of CCR5 was decreased compared to that observed in cells that were not stimulated. CCR5 down regulation was more marked in CD57+ memory CD4+ T cells than in non-CD57− memory CD4+ T cells. D.) PBMC were stimulated with CD2+, CD3+, CD28+ beads in the absence of BFA with either no anti-MIP-1α or anti-MIP-1β blocking antibody or 2, 5 or 10 µg of anti-MIP-1α and anti-MIP-1β/ml. At the conclusion of 5 h incubations containing CD2+, CD3+, CD28+ beads and no BFA the frequency of surface expression of CCR5 was greater than in incubations containing anti-MIP-1α and anti-MIP-1β blocking antibodies than in matched incubations not containing blocking antibodies. CCR5 surface expression for both CD57+ and CD57− memory CD4+ T cells are shown as a function of the amount of blocking antibodies in each incubation. The solid line and dashed line represent CCR5 expression of unstimulated CD57− and CD57+ memory CD4+ T cells, respectively. (0.67 MB TIF) [file ppat.1000646.s002.tif]

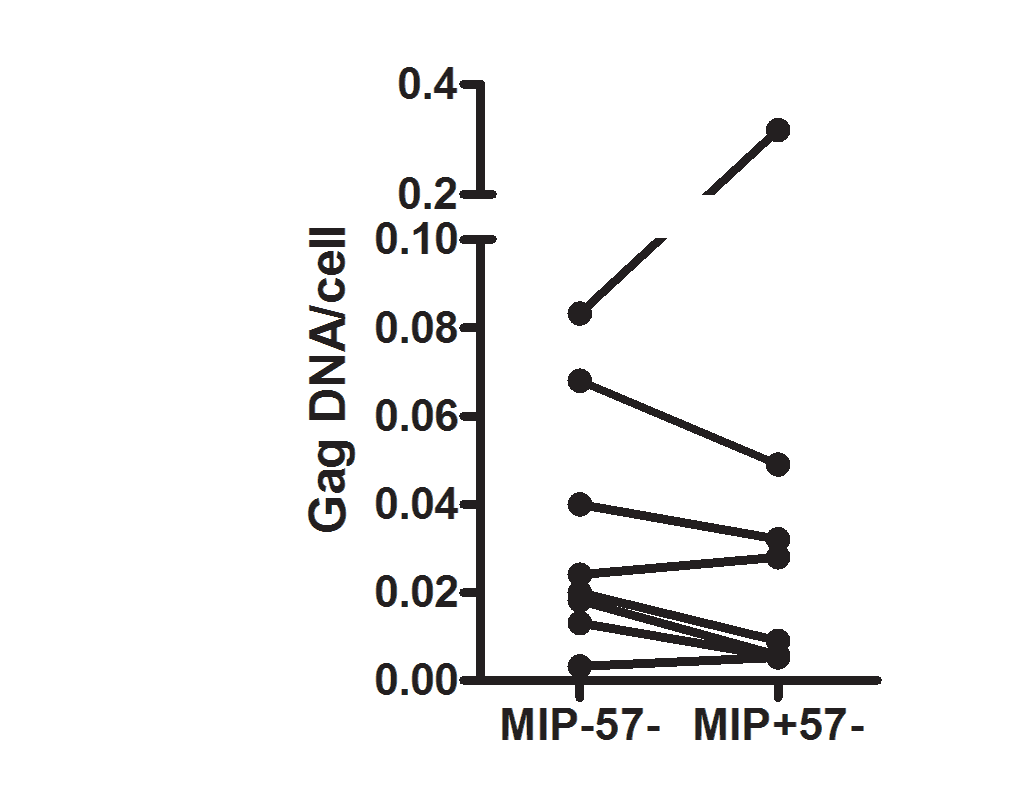

Supplement: Figure S3 — SEB stimulated CD57− memory CD4+ T cells that produce MIP-1β do not contain lower amounts of cell associated Gag DNA than do no non-MIP-1β producing cells CD57− memory CD4+ T cells. PBMC at a concentration of 3E06 PBMC/ml were incubated in the presence of BFA and 1µg/ml SEB. At the end of a 6h incubation period cells were stained and sorted in the same manner as shown in Figure 6. Unlike CMV-specific CD4+ T, no significant difference was observed in cell associated Gag DNA in IFNγ producing CD57− cells which produced MIP-1β and those that did not. (0.10 MB TIF) [file ppat.1000646.s003.tif]
